# Noninvasive estimation of pulmonary outflow tract obstruction: a comparative study of phase contrast CMR and Doppler echocardiography versus cardiac catheterization

**DOI:** 10.1186/1532-429X-17-S1-Q105

**Published:** 2015-02-03

**Authors:** Johannes T Kowallick, Michael Steinmetz, Andreas Schuster, Christina Unterberg-Buchwald, Thuy T Nguyen, Martin Fasshauer, Wieland Staab, Olga Hösch, Christina Rosenberg, Thomas Paul, Joachim Lotz, Jan M Sohns

**Affiliations:** 1 Institute for Diagnostic and Interventional Radiology, University Medical Centre Göttingen, Georg-August-University Göttingen, Göttingen, Germany; 2 DZHK (German Centre for Cardiovascular Research), partner site Göttingen, Göttingen, Germany; 3 Department of Pediatric Cardiology and Intensive Care Medicine, University Medical Centre Göttingen, Göttingen, Germany; 4 Department of Cardiology and Pneumology, University Medical Centre Göttingen, Göttingen, Germany

## Background

Historically, the catheter peak-to-peak pressure gradient (PPG) has been used as the diagnostic gold standard to evaluate the degree of pulmonary outflow tract obstruction in congenital heart disease (CHD) and was employed to decide when to intervene. Today, estimated maximal Doppler gradients are generally decisive. Cardiovascular phase contrast magnetic resonance (PCMR) measurements are frequently performed during routine follow-up. However, it remains unclear how to deal with PCMR flow velocities that can also serve for the estimation of pressure gradients.

## Methods

In 75 patients with pulmonary outflow tract obstruction maximal and mean PCMR gradients were compared to maximal and mean Doppler gradients. Additionally, in a subgroup of 31 patients maximal and mean PCMR and Doppler pressure gradients were compared to catheter PPG.

## Results

Maximal and mean PCMR gradients underestimated pulmonary outflow tract obstruction as compared to Doppler (maximal PCMR: bias = +8.4 mmHg, r = 0.89, p < 0.001; mean PCMR: +4.3 mmHg, r = 0.88, p < 0.001). However, in comparison to catheter PPG, maximal PCMR gradients and mean Doppler gradients revealed best agreement (maximal PCMR: bias = +1.8 mmHg, r = 0.90, p = 0.14; mean Doppler: bias = -2.3 mmHg, r = 0.87, p = 0.17). Mean PCMR gradients underestimated, while maximal Doppler gradients systematically overestimated catheter PPG (mean PCMR: bias = -7.7 mmHg, r = 0.90, p < 0.001; maximal Doppler: bias = +13.9 mmHg, r = 0.88, p < 0.001).

## Conclusions

Estimated maximal PCMR pressure gradients and mean Doppler gradients from routine CHD follow-up agree well with invasively assessed PPG. There is evidence to either apply maximal PCMR gradients or mean Doppler gradients (instead of maximal Doppler gradients) to evaluate the severity of pulmonary outflow tract obstruction during follow-up of CHD.

## Funding

N/A.

